# Do Individual and Joint Action Goals Modulate Imitative Response Tendencies?

**DOI:** 10.5334/joc.483

**Published:** 2026-01-09

**Authors:** Maximilian Marschner, Günther Knoblich, David Dignath

**Affiliations:** 1 Department of Cognitive Science, Central European University, Vienna, Austria; 2 Department of Psychology, Eberhard Karls University of Tübingen, Tübingen, Germany

**Keywords:** Social Interaction, Imitation, Joint Action, Action Goals, Action Observation

## Abstract

Coordinated social interaction requires people to control their tendencies to imitate each other’s actions. Previous research suggests that imitative response tendencies become modulated by the goals to which one’s own and others’ actions are individually or jointly directed. However, an open question is how different levels of goal representation (ranging from higher-level goals that specify joint or individual action outcomes to lower-level goals encoding own and others’ movement features) interact and shape imitative congruency effects during social interactions. To address this gap, we conducted two online experiments, in which participants selected one of two action targets in turn with a virtual co-actor to achieve either individual or joint task goals. We manipulated imitative congruency between both task partners’ task contributions regarding their individual action goals as well as their lower-level movement goals. Our results showed that participants’ task performance was driven by imitative congruency between their own and their partner’s individual action goals, which modulated effects of imitative congruency between their own and their partner’s low-level movement goals. Interestingly, these imitation effects were found to be present regardless of instructing participants to work towards individual or joint task goals. While supporting goal-directed theories of imitation, our findings suggest that modulations of imitative response tendencies may stem from domain-general action planning and control processes that operate across social and non-social task settings, and that instructions to pursue joint rather than individual task goals exert only limited influence on imitative action tendencies in interactive task contexts.

## 1. Introduction

To engage in social interactions, people need to interpret the actions they observe in others and produce appropriate and timely responses to them. Understanding how these processes work in concert and enable interaction partners to coordinate their actions is a central question for research on joint action (Sebanz et al., 2006). A common assumption in this field of research is that people process others’ actions through the lens of their own action capacities, i.e., that they reuse cognitive resources deployed for action execution to simulate and predict the actions they perceive in others (Wilson & Knoblich, 2005; Wolpert et al., 2003). This idea has gained ample empirical support from neuroimaging studies showing that action observation and action execution recruit similar brain circuits (for a review see e.g., Rizzolatti & Sinigaglia, 2016) as well as from behavioural research showing that people tend to imitate the actions they perceive in others (Heyes, 2011; c.f., Genschow & Cracco, 2025 for an exhaustive collection of relevant research findings). This imitative response tendency is indexed by the well-replicated finding that actions are initiated faster while observing similar (i.e., congruent) compared to dissimilar (i.e., incongruent) actions of somebody else (imitative congruency effect; see Cracco et al., 2018 for a meta-analysis). Prominent theoretical accounts propose that this imitative congruency effect is the result of a direct matching process between observed and self-executed movement features, suggesting that action observation automatically and obligatorily activates corresponding motor representations in the observer (e.g., Heyes, 2011; Iacoboni et al., 1999; Rizzolatti et al., 2001).

Direct matching between perceived and self-executed actions can support social interactions by allowing interaction partners to predict and align their actions (Knoblich et al., 2011). However, it also poses challenges for social interactions that require interaction partners to perform dissimilar yet complementary actions to achieve their goals (Sartori & Betti, 2015). For instance, handing someone a glass of champagne requires well-coordinated, yet different ways of grasping the glass by both interaction partners. Arguably, in these situation, automatic imitation of observed movements would interfere with task performance, raising the important question of whether and how our cognitive system can modulate imitative response tendencies in a flexible and context appropriate way (Brass & Heyes, 2005).

A possible solution to this problem is offered by goal-directed theories of imitation. According to these accounts, imitation is not restricted to the faithful copying of others’ bodily movements, mediated by an automatic matching process between observed and self-executed movement features. Instead, goal-directed accounts construe imitation as an interpretative process in which individuals infer and reproduce the *goals* underlying others’ actions (e.g., Bekkering et al., 2000; Csibra, 2007; Gattis et al., 2002; Wohlschläger et al., 2003; c.f., Heyes, 2013 for discussion). Central to these accounts is the assumption that actions can be represented at different, hierarchically organized levels of abstraction, ranging from overarching *task goals* (e.g., building a tower) over more proximal *action goals* (e.g., placing a brick onto another) to specific *movement goals* (e.g., lifting one’s right arm) (c.f., Grafton & Hamilton, 2007; Kilner et al., 2007; Pacherie, 2008; Uithol et al., 2012; Vallacher & Wegner, 2012). Goal-directed accounts of imitation assume that observers prioritize the reproduction of higher-level goals over lower-level movement patterns, such that tendencies to imitate low-level movement features of observed actions can become overruled by interpretations of observed actions in terms of their higher-level task and action goals (Ondobaka & Bekkering, 2012, 2013).

### The influence of action goals on imitation

While a growing body of evidence supports goal-directed theories of imitation, whether — and if so how — higher-level goal representations modulate imitative response tendencies still remains poorly understood. Preliminary evidence has been provided by studies showing that interference effects between perceived and executed *movement goals* are susceptible to imitative congruency relations between own and others higher-level *action goals* (Massen & Prinz, 2007; Ondobaka et al., 2012, 2013). E.g., a seminal study by Ondobaka et al. (2012) showed that imitative congruency effects between own and other’s movement goals (e.g., arm reaching movements to the left or the right) emerge only if co-actors perform their movements to achieve congruent action goals (e.g., selecting similar action targets), but not if co-actors perform their movements to achieve incongruent action goals (e.g., selecting dissimilar action targets). Moreover, performing actions towards congruent compared to incongruent action goals was shown to benefit participants’ response performance overall, indicating that people have a tendency to imitate higher-level action goals of others independent of the means they choose to reach them.

These findings indicate a central role of action goals in shaping imitative congruency effects during social interactions and suggest a hierarchical control mechanism for imitative behaviour in which congruency relations between observed and self-executed actions at higher levels (e.g., action goals) modulate imitative congruency effects at lower-levels of action representation (e.g., movement goals). However, this conclusion has been challenged by recent studies that failed to provide conclusive evidence for a modulating role of action goals on imitative congruency effects between own and others’ lower-level movement goals (Cole et al., 2018; Janczyk et al., 2016). As such, the extent and conditions under which higher-level action goals become able to modulate imitative congruency effects between lower-level movement goals still remains a subject of ongoing debate (see Bird et al., 2007; Chiavarino et al., 2013; Leighton et al., 2010; Longo et al., 2008; Wild et al., 2010 for related discussions on the role of goals in imitation).

### The influence of joint task goals on imitation

Further evidence for the notion that higher-level goal representations modulate imitative response tendencies in social interactions comes from research on joint action (see McEllin et al., 2018 and Sebanz & Knoblich, 2021 for reviews). As mentioned above, joint action often requires people to perform actions that are incongruent yet complementary to those of their co-actors (Sartori & Betti, 2015), making joint action an interesting test case for the flexibility of imitative response tendencies. Initial evidence for modulations of imitative response tendencies in joint action contexts has been provided by a range of studies showing that explicit instructions and implicit requests to *complement* rather than to imitate observed actions of an interaction partner can reduce or even reverse imitative congruency effects between own and other’s movement goals (Bardi et al., 2015; Betti et al., 2019; Newman-Norlund et al., 2007; Ocampo et al., 2011; Ocampo & Kritikos, 2010; Poljac et al., 2009; Sartori et al., 2012, 2013; van Schie et al., 2008). Going beyond research on the role of individual action goals in modulating imitative congruency effects described above, these findings suggest a special role for goal representations that encode the joint outcomes of co-actors’ complementary action contributions, i.e., what they are achieving together as a group (see Butterfill, 2012 and Sacheli et al., 2015). We refer to this level of goal representation as *joint task goals*.

Crucially, direct support for the notion that joint task goals shape imitative response tendencies has been provided by recent studies showing that imitative congruency effects between own and others’ movement goals are indeed reduced (Clarke et al., 2019; Rocca et al., 2023; Schmitz et al., 2023) or even absent (Sacheli et al., 2018, 2019) when own and others’ movements are performed as interrelated contributions towards a joint goal. To explain these findings, theoretical models of joint action propose that representations of joint task goals lead co-actors to integrate representations of their own and their partners’ individual action contributions into hierarchical joint action plans that encode relational constrains between co-actors’ individual action and/or movement goals (Candidi et al., 2015; Clarke et al., 2019; Pesquita et al., 2018; Sacheli et al., 2018; Sinigaglia & Butterfill, 2022; Zapparoli et al., 2022).

### The present research

In light of the reviewed findings, we set out to further investigate if and how higher-level goal representations modulate imitative response tendencies during social interactions. Our motivation to do so was two-fold. First, we thought to re-assess previous research presenting mixed evidence for the notion that individual action goals modulate imitative congruency effects between own and others’ lower-level movement goals. To this end, we conducted two conceptual replications of the study by Ondobaka et al. (2012) in which we manipulated imitative congruency relations between the actions of two co-actors at the level of their individual action and movement goals. Second, building on previous indications that joint task goals reserve a dedicated role for coordinating co-actors’ individual contributions to a social interaction (Clarke et al., 2019; Rocca et al., 2023; Sacheli et al., 2018), we further asked whether and how *joint* as opposed to *individual* task goals add to the hierarchical control of imitative response tendencies in social interactions. To do so, in both experiments, we further instructed different groups of participants to either work towards individual or joint task goals, testing whether joint task goals might further modulate imitative congruency effects between co-actors action and movement goals.

In two experiments, participants performed a sequential target selection task together with a virtual partner, modelled after Ondobaka et al. (2012). On each trial, participants first observed a virtual partner (in the following referred to as the co-actor) selecting the higher or the lower of two playing cards (Experiment 1) or line drawings (Experiment 2) and where required to select one of two playing cards or line drawings themselves in response. Participants target selection was based on condition specific task instructions that required participants to select either the higher or the lower of their targets respectively (we refer to this as participants’ action goal). Participant’s and their co-actor’s action targets were presented side by side, and participants selected their respective target on a given trial by pressing a left or a right key on their keyboard to select the left or the right of their targets respectively (we refer to this as participants’ movement goal). In both experiments, we manipulated imitative congruency between co-actors’ action goals (i.e., whether participants had to select the higher or the lower of their targets after observing their co-actor selecting the higher or lower of their targets) and between co-actors’ movement goals (i.e., whether participants had to select the left or the right of their targets after observing their co-actor selecting the left or the right of their targets).

Deviating from the original study by Ondobaka et al. (2012), congruency between co-actors’ action goals was manipulated between- rather than within-subjects to rule out possible conflict or carry over effects between the different task instructions. Moreover, presenting the targets of both co-actors side by side deviates from the studies by Cole et al. (2018), Janczyk et al. (2016) and Ondobaka et al. (2012) in which co-actors’ action targets were presented opposite to each other, so that the their absolute spatial locations overlapped. In these setups, imitative congruency effects at the level of co-actors movement goals (e.g., faster selection of left targets after observing one’s partner responding to their left compared to their right target) have been attributed to effects of attentional disengagement from target locations cued by previous partner responses (referred to as *social inhibition of return*, see Cole et al., 2019 for review). By presenting co-actors’ action targets side by side, our setup eliminates spatial overlap between co-actors’ absolute target locations, ensuring that imitative congruency effects between co-actors’ movement goals can be attributed to automatic imitation (which are the focus of the present study) rather than to purely attentional mechanisms.

Crucially, further extending the experimental design by Ondobaka et al. (2012), task instructions for selecting an action target on a given trial was manipulated between two differed groups of participants (we refer to this as participants’ task goal). While one group of participants was explicitly instructed to always match or mismatch the action goal of their co-actor by selecting the higher or the lower of their targets (individual task goal condition), another group of participants was instead instructed to complement the card selection of their co-actor to achieve a joint outcome that specified specific relations between their individually selected action targets (joint task goal condition).

Based on previous research by Ondobaka et al. (2012, 2013), for the individual task goal condition, we expected to find imitative congruency effects at the level of co-actors’ individual action goals as well as at the level of their lower-level movement goals. More specifically, in line with the idea of an hierarchical control mechanism for imitative behaviour, congruency relations between co-actors’ individual action goals should modulate imitative congruency effects between co-actors’ movement goals. Replicating this modulation effect in the current study would settle concerns about the modulating role of higher-level action goals on imitative response tendencies raised by recent studies (Cole et al., 2018; Janczyk et al., 2016). Furthermore, based on research suggesting a dedicated role of joint task goals in modulating imitative response tendencies in social interactions (Clarke et al., 2019; Rocca et al., 2023; Sacheli et al., 2018), we tested whether imitative congruency effects between co-actors’ individual action and movement goals would be further modulated by the joint task goal to complement another person’s action in the service of a joint goal. In particular, based on the idea that joint task goals modulate the impact of individual action and movement goals on imitative behaviour, we hypothesized that imitative congruency effects at the level of co-actors’ individual action and movement goals would be reduced or even absent in the joint task goal condition.

## 2. Experiment 1

### 2.1. Methods

#### 2.1.1. Transparency and openness

Data for Experiment 1 was collected in April 2023. Raw data, analysis scripts, stimuli, and experiment scripts are accessible in an Open Science Framework project repository (https://osf.io/wc96q/?view_only=dd5091015d744e81a0295afdb0050d49). The study design, hypotheses, sampling plan and data exclusion criteria were preregistered in the Open Science Framework. The preregistration is accessible under https://osf.io/5dn47/?view_only=04880f3bbccf4e3ba8c7bda9f5eca11f.

#### 2.1.2. Participants

Two considerations determined our target sample size. First, we powered the experiment to replicate the action goal congruency effect reported by Ondobaka et al. (2012) in a between-subjects design. Therefore, Experiment 1 was powered to detect a medium to large sized effect of *d* = 0.6 in a two-samples *t*-test with 80% power at an alpha level of .05 (two-tailed). Conducting an a priori power analysis in G*Power (Faul et al., 2007) with these input parameters, we calculated a minimum sample size of 45 participants per group. Note that 45 participants per group also yields sufficient power to detect a crossed interaction pattern between action and movement goal congruency in our experimental design (see simulation results by Brysbaert, 2019). Second, since we were not aware of any published research that could guide power calculations for a possible modulation of imitation effects between the individual and the joint task goal condition, we decided to adopt the above number of participants per group for the joint task goal condition as well, leading to a total target sample size of *N* = 180. Conducting a sensitivity analysis in G*Power showed that this sample size would be sufficient to detect an interaction effect between the two between-subjects factors in our design of d = 0.42 with 80% power at an alpha level of .05. To account for potential exclusion of participants, we eventually decided to collect data from 200 participants, split into 50 participants per group of our 2 × 2 between-subjects experimental design (see below). Data of eight participants were removed (see preregistered exclusion criteria), so that our final sample size included *N* = 192 participants (mean age = 30.8, SD = 9.2; 49% female, 51% male). Participants were recruited on Prolific (https://www.prolific.com). All participants had normal or corrected-to-normal vision and hearing and were fluent in English. All participants gave written informed consent and received monetary compensation for their participation (2 GBP for a total duration of approx. 12 minutes). Ethical approval for the study was granted by the Psychological Research Ethics Board (PREBO) of Central European University.

#### 2.1.3. Stimuli and apparatus

Participants completed the experiment online in a browser on their own personal computer. Experiment files and data were stored on a commercial server provider for psychological experiments (https://www.pavlovia.org). Stimuli presentation and response recording was controlled by a custom made script written in the jsPsych JavaScript framework (de Leeuw et al., 2023).

**Figure 1:**
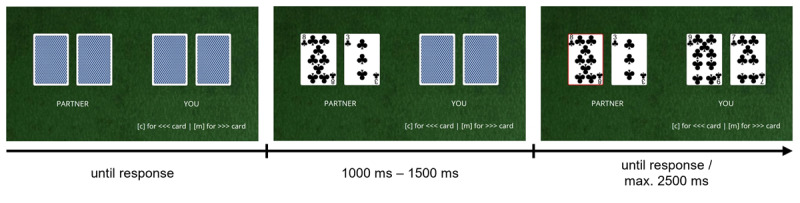
Trial timeline and stimuli example for Experiment 1. In the individual task goal condition, participants were instructed to either match or mismatch the card selection of their co-actor (indicated by the red frame) by selecting the higher or the lower of their cards. I.e., in the example above, half of the participants had to select the higher of their cards (clubs nine) while the other half had to select the lower of their cards (clubs seven) after observing their co-actor selecting the higher of their cards (clubs eight). In the joint task goal condition, participants were instead instructed to select one of their cards that complemented an ascending or a descending number sequences together with the card selected by their co-actor. Here, one half of the participants was instructed to complement an ascending sequence when their partner selected the higher of their cards and to complement a descending sequence when their partner selected the lower of their cards (i.e., selecting clubs nine as the successor of clubs eight in the example above), while the other half was instructed to complement an ascending sequence when their partner selected the lower of their cards and to complement a descending sequence when their partner selected the higher of their cards (i.e., selecting clubs seven as the predecessor of clubs eight in the example above). Note that selecting the correct card in each condition could require participants to select the card at the same or the opposite relative spatial position as their co-actor (i.e., selecting the left or the right of their cards after the co-actor selected the left of their cards).

Participants were presented with four horizontally aligned playing cards, displayed against a green background (see Figure 1). The cards were drawn from a subset of a standard card deck comprising the numerical card values of the clubs’ suit in the range from two to nine. Two of the cards were assigned to a virtual partner and were presented on the left half of the screen. They always showed one out of six possible card combinations from the card set, containing card values in the range from three to eight with a numerical distance between cards larger than two (i.e., [3|6], [3|7], [3|8], [4|7], [4|8], [5|8]). The other two cards were assigned to the participants and were presented on the right half of the screen. Importantly, they always showed the next higher and the next lower card value relative to the card selected by the co-actor on a given trial (e.g., if the co-actor selects clubs’ eight, the two cards of the participants were clubs’ seven and clubs’ nine). Participants selected the left or the right of their assigned cards by pressing the “c” (left) and the “m” (right) key on their keyboard respectively. Figure 1 depicts an example arrangement of the four playing cards on an individual trial.

#### 2.1.4. Procedure and design

Each trial started with the presentation of all four playing cards faced down. Participants started a trial by pressing the space bar. First, the two cards of the co-actor were revealed. After a variable delay (1000 ms–1500 ms), one of the co-actor’s cards was highlighted with a red frame that indicated their card selection and the two cards of the participant were revealed. Participants then had to select one of their cards as quickly as possible within a maximum response window of 2500 ms. If participants gave a response, their selected card was highlighted by a red frame for 500 ms and participants received feedback (displayed for 2000 ms) informing them whether they had selected the correct card (“correct”), the wrong card (“error”) or had failed to respond within the response window (“too slow”). The next trial started after a blank screen displayed for 500 ms. The trial sequence is illustrated in Figure 1.

The rule that specified which card participants had to select on a given trial differed between four experimental groups. In the individual task goal condition, participants were instructed to always “match” or to always “mismatch” the card selection of the co-actor by selecting the higher or the lower of their cards. Thus, half of the participants in the individual task goal condition had to adopt the same (i.e., congruent) action goal as the co-actor (e.g., if the co-actor selected the higher of their cards, participants had to select the higher of their cards too) while the other half had to adopt the opposite (i.e., incongruent) action goals as the co-actor (e.g., if the co-actor selected the higher of their cards, participants had to select the lower of their cards instead).

In contrast, in the joint task goal condition, participants were instructed to complement the card selection of the co-actor by selecting one of their cards that completed an ascending or a descending number sequences together with the card selected by the co-actor. In particular, half of the participants in the joint task goal condition were instructed to complement an *ascending* number sequence when the co-actor selected the *higher* of their cards and to complete a *descending* number sequence when the co-actor selected the *lower* of their cards. The other half of participants in the joint task goal condition was instead instructed to complete an *ascending* number sequence when the co-actor selected the *lower* of their cards (e.g. if the co-actor selects the clubs three card, participants had to select the clubs four card) and to complete a *descending* number sequence when the co-actor selected the *higher* of their cards (see Figure 1 for examples).^1^

Importantly, the instructions for the two groups in the joint task goal conditions ensured that one group had to select the higher (or lower) of their cards when the co-actor selected the higher (or the lower) of their cards, while the other group had to select the higher (or lower) of their cards when the co-actor selected the lower (or higher) of their cards. Thus, although not relevant for performing the task as instructed, participants in the joint task goal condition had to select one of their cards by *implicitly* adopting the same (i.e., congruent) or opposite (i.e., incongruent) action goal as the co-actor. As such, the individual and the joint task goal condition were procedurally and perceptually identical and only differed in terms of either explicitly emphasizing participants’ *individual* action goals (selecting the higher or the lower of their cards) or rather emphasizing co-actors’ *joint goal* (complementing the card selection of the co-actor to build ascending or a descending number sequences).^2^ In addition, to highlight the joint outcome of co-actors’ individual card selections, only the selected card of the partner and the selected card of the participant remained on screen during feedback display in the joint task goal condition, while in the individual task goal conditions all four cards remained on screen until the end of the trial.

For all experimental groups, congruency between movement goals was manipulated by varying the relative spatial position of the assigned cards, so that selecting the correct card on a given trial required participants to select either the left or the right of their cards after observing their co-actor selecting the left or the right of their cards respectively.

Thus, the study had a 2 (Task Goal: Individual vs. Joint) × 2 (Action Goal Congruency: Congruent vs. Incongruent) × 2 (Movement Goal Congruency: Congruent vs. Incongruent) mixed factorial design, with Task Goal and Action Goal Congruency manipulated between and Movement Goal Congruency manipulated within subjects. After going through a self-paced instruction phase explaining the trial structure and the condition specific card selection rules, participants performed eight training trials that were randomly drawn from a set of forty-eight possible card arrangements (counterbalanced for Movement Goal Congruency). If participants responded correctly on more than four trials during training,^3^ they proceeded to the main part of the experiment and performed forty-eight test trials in their assigned experimental condition appearing in fully randomized order (Movement Goal Congruency was counterbalanced across trials).

#### 2.1.5. Data analysis

Training trials were excluded from analysis. Test trials with response omissions were removed prior to analysis (0.9% of all trials). For the remaining test trials, error rates (ER, relative frequency of wrong card selections) and response times for correct responses (RTs) were analysed by means of separate 2 (Task Goal: Individual vs. Joint) × 2 (Action Goal Congruency: Congruent vs. Incongruent) × 2 (Movement Goal Congruency: Congruent vs. Incongruent) mixed ANOVAs with Task Goal and Action Goal Congruency as between-subjects factors and Movement Goal Congruency as within-subjects factor. For all inference statistical analysis, the alpha level was set to .05. Significant interactions were followed up by analysis of simple main effects that were Bonferroni corrected for multiple comparisons.

To supplement the results of our preregistered frequentist analysis, we also conducted an exploratory Bayesian ANOVA using JASP (JASP Team, 2024). This allowed us to calculate Bayes factors to assess the relative evidence in the data for different factor models. To quantify the evidence for individual main and interaction effects of the design factors, we report model-averaged inclusion Bayes factors (BF_Inclusion_), which measure the strength of evidence for including a particular factor or interaction in a model compared to models where that factor or interaction is excluded (see van den Bergh et al., 2020). Additionally, we calculated Bayes factors for individual model comparisons, assessing the relative evidence for different combinations of factors in explaining participants’ performance. Therefore, we report Bayes factors (BF_01_) quantifying the relative evidence of individual factor models against the best fitting model of our design factors (cf. van den Bergh et al., 2020). We used the default specification of prior distributions for parameter values in each model offered in JASP (cf. van den Bergh et al., 2020) set to its default options (*r* scale for fixed effects = .5). To assess the robustness of the results, we repeated the analysis with two different prior specifications (*r* scale for fixed effects = .2 and *r* scale for fixed effects = .8). Prior probabilities for individual models were drawn from a uniform distribution.

### 2.2. Results

**Figure 2:**
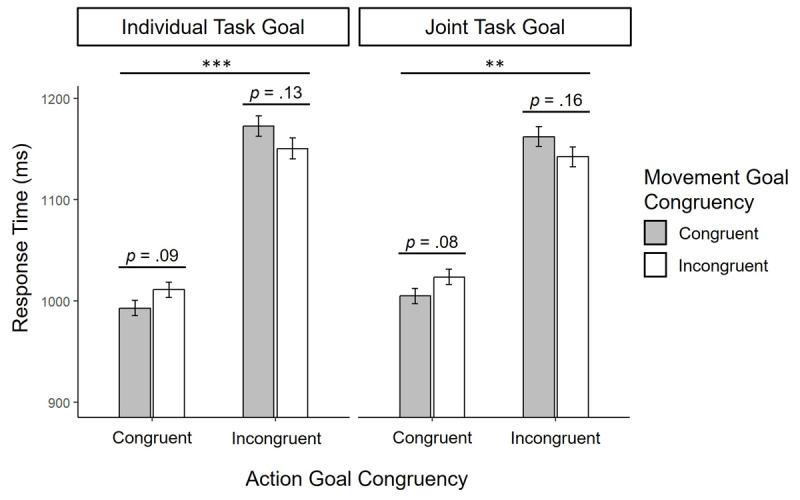
Mean response times in Experiment 1 as a function of Task Goal, Individual Goal Congruency and Response Congruency. Error bars represent standard errors of the mean (SEM) corrected for within subjects designs (Morey, 2008). Asterisks denote significant simple-effects contrasts between factor levels, conditional on the remaining factors; numeric labels indicate exact p-values for non-significant contrasts. ***p < .001, **p < .01.

The results of the RT analysis of Experiment 1 is depicted in Figure 2.

The RT analysis revealed a significant main effect of Action Goal Congruency, *F*(1, 188) = 23.1, *p* < .001, η_p_^2^ = .11, BF_Inclusion_ = 3413.9, with faster RTs for participants in the action goal congruent (mean = 1008 ms, 95% CI [965 ms, 1050 ms]) compared to the action goal incongruent conditions (mean = 1156 ms, 95% CI [1113 ms, 1200 ms]). Importantly, the interaction between Action Goal Congruency and Task Goal was non-significant (*F* < 1, BF_Inclusion_ = 0.527), thus providing no evidence that the main effect of Action Goal Congruency was moderated by participants’ Task Goal (Individual vs. Joint). Yet, results showed a significant interaction between Action Goal Congruency and Movement Goal Congruency, *F*(1, 188) = 10.1, *p* = .002, η_p_^2^ = .05, BF_Inclusion_ = 14.43. Follow-up analysis showed that this interaction was driven by faster RTs in movement goal congruent trials (mean = 998 ms, 95% CI [988 ms, 1009 ms]^4^) compared to movement goal incongruent trials (mean = 1017 ms, 95% CI [1007 ms, 1028 ms]) when co-actors’ action goals were congruent to each other, *F*(1,97) = 6.17, *p_Bonf_* = .04, η_p_^2^ = .06, and descriptively slower RTs in movement goal congruent trials (mean = 1168 ms, 95% CI [1154 ms, 1182 ms]) compared to movement goal incongruent trials (mean = 1147 ms, 95% CI [1133 ms, 1160 ms]) when co-actors’ action goals where incongruent to each other, *F*(1,93) = 4.45, *p_Bonf_* = .08, η_p_^2^ = .05. The absence of a significant three-way interaction between Action Goal Congruency, Movement Goal Congruency and Task Goal (*F* < 1, BF_Inclusion_ = 0.319), showed no evidence that this interaction pattern was moderated by participants’ Task Goal (Individual vs. Joint). All other effects were non-significant (all *F* < 1).

In line with the frequentist analysis, Bayesian model comparison revealed that the factor model including main effects of Action and Movement Goal Congruency as well as their interaction provided the best fit to the data (BF_M_ = 9.2). Importantly, this model was preferred over the model that further included the interaction between Task Goal and Action Goal Congruency (BF_01_ = 3.96), quantifying substantial evidence against a moderating influence of Task Goal on the effect of Action Goal Congruency. Similarly, the model including only main effects for Action and Movement Goal Congruency as well as their interaction, was strongly preferred over the model that included further interactions up to the three-way interaction between all design factors (BF_01_ = 91.89), thus quantifying strong evidence that the interaction pattern between Action and Movement Goal Congruency was not moderated by participants’ Task Goal. A table with all Bayesian model comparisons is presented in Appendix A. Using different prior specifications for parameter values in each model produced qualitatively similar result patterns (result tables for Bayesian model comparisons with different prior options can be found in the supplement).

Results of the ER analysis were largely congruent with the RT analysis and there was no indication for a speed-accuracy trade-off. The detailed results of the ER analysis are presented in Appendix A.

### 2.3. Discussion

The results of Experiment 1 revealed three main findings. First, we found an imitative congruency effect at the level of co-actors’ action goals: Participants responded faster if their action goal (selecting the higher or the lower of their cards) was congruent (vs. incongruent) to the action goal of their co-actor. Second, we found a significant interaction between action and movement goal congruency: Congruent (vs. incongruent) movement goals (e.g. participants selecting their left card after the co-actor selected their left card) only led to faster responses when co-actors action goals were congruent but not when they were incongruent to each other. These two findings replicate those of Ondobaka et al. (2012) and support the notion that imitative response tendencies are primarily determined by congruency relations between co-actors’ higher-level action goals which modulate the influence of congruency relations between co-actors’ lower-level movement goals. Thus, our findings support goal-directed theories of imitation by showing that interpretations of observed actions in terms of their higher-level action goals modulate imitative congruency effects between lower-level representations of own and others’ actions.

Third, however, we found no evidence that the effects of action and movement goal congruency were dependent on participants’ task goal to either match or mismatch the card selection of the co-actor (individual task goal condition) or to complement the card selection of the co-actor to achieve a joint goal (joint task goal condition). More specifically, we found no indications that instructing participants to complement the card selection of their co-actor to achieve a joint outcome helped participants to overcome effects of imitative congruency between their own and their co-actor’s individual action and movement goals. This finding speaks against the hypothesis that joint task goals add to the hierarchical control of imitation by modulating imitative congruency effects between co-actors’ individual action and/or movement goals.

Yet, it is possible that the missing influence of task goal instructions on imitative congruency effects in Experiment 1 could be explained by overall task difficulty in the joint task goal condition. In particular, we reasoned that participants in the joint task goal condition could have faced difficulties implementing the task instructions to build ascending and descending number sequences together with their co-actor and reverted to perform the task with the individual task goal of matching or mismatching the card selection of their co-actors instead. Reconceptualization of task goals in the face of increasing task difficulty is a central assumption of action identification theory which suggests that increasing difficulties to perform an action under a current task conceptualization leads people to conceptualize their task in alternative ways that ensure more efficient performance (Vallacher & Wegner, 1987, 2012).

Based on this speculation, we conducted a second experiment, closely replicating Experiment 1 with a different set of stimuli as action targets, that should reduce overall task difficulty and hence might facilitate participants’ encoding of their joint goal in the joint task goal condition.

## 3. Experiment 2

Experiment 2 provided a close replication of Experiment 1 with a different set of target stimuli: Instead of playing cards with different numerical values, participants and their co-actor selected between line drawings that differed in their relative height. While Experiment 1 required participants to process abstract symbolic information to discriminate between action targets (numerical differences between symbolic card values), target selection in Experiment 2 required participants to process concrete perceptual information instead (perceptual height differences between line drawings). Thus, action selection in Experiment 2 could build on modal representations of target stimuli which are thought to be processed more efficiently in contrast to amodal representations involved in the processing of more abstract symbolic information (Kaup et al., 2024).

Changing the modality of stimulus representations should thus enable more efficient processing of magnitude relations between action targets, lowering overall task difficulty in Experiment 2 compared to Experiment 1. We hypothesized that this might help participants in the joint task goal condition to select their targets in relation to the joint task goal of complementing their co-actors target selection, which could make possible modulations of joint task goals on imitative congruency effects more pronounced.

### 3.1. Methods

#### 3.1.1. Transparency and openness

Data for Experiment 2 was collected in April 2023. Raw data, analysis scripts, stimuli, and experiment scripts are accessible in the Open Science Framework project repository referred to above. Hypotheses, study design, sampling and analysis plan of Experiment 2 were identical to Experiment 1, thus following the same preregistration protocol referred to above.

#### 3.1.2. Participants

A new sample of 200 Prolific users took part in Experiment 2, randomly assigned to the same experimental conditions as in Experiment 1. From this sample, twelve participants met the preregistered exclusion criteria and were dropped from analysis, leading to a final sample size of *N* = 188 (mean age = 28.3, SD = 8.0; 36% female, 64% male).

#### 3.1.3. Stimuli and apparatus

**Figure 3:**
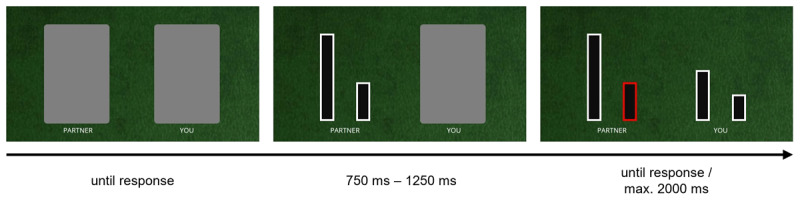
Trial timeline and stimuli example for Experiment 2. Task instructions for the different experimental conditions were analogous to those of Experiment 1.

In Experiment 2, instead of playing cards, participants were presented with four horizontally aligned line drawings (see Figure 3). The line drawings were drawn from a set of eight line drawings with equal width but different relative heights. From the lowest to the highest line drawing of the stimulus set, line height increased by one unit equal to the size of the lowest line. From this set, two lines were assigned to the co-actor and were presented on the left half of the screen. They always depicted one out of six possible line combinations from the stimulus set containing only lines from the second lowest to the second highest and only those line combinations with a height difference of at least two units equal to the height of the lowest line. The other two lines were assigned to the participants and were presented on the right half of the screen. Importantly, they always showed the next higher and the next lower line relative to the line selected by the co-actor on a given trial. Participants selected the left or the right of their assigned lines by pressing the “c” (left) and “m” (right) key on their keyboard respectively. Figure 3 depicts an example arrangement of the four line drawings on an individual trial.

#### 3.1.4. Procedure and design

The procedure and design of Experiment 2 were identical to that of Experiment 1, despite the following differences. At the beginning of each trial, two grey boxes covered the lines assigned to the participant and their co-actor. After starting a trial by pressing the space bar, the left box disappeared and revealed the two lines of the co-actor. The co-actor’s selection was indicated by a red frame surrounding one of their lines appearing 750 ms to 1250 ms after stimulus onset. Together with the selection of the co-actor, the right box disappeared and revealed the two lines assigned to the participant. Participants then had up to 2000 ms to select one of their lines. Timing of response feedback was identical to Experiment 1. The trial procedure is depicted in Figure 3.

Parallel to Experiment 1, the rule that specified which line participants had to select on a given trial differed between four experimental groups. In the individual task goal condition, one group of participants was instructed to always “match”, while another group of participants was instructed to always “mismatch” the line selection of the co-actor by selecting the higher or the lower of their lines. In the joint task goal condition on the other hand, one group of participants was instructed to select the line that produced an *ascending* (a descending) sequence with the line selected by the co-actor when the co-actor selected the *higher* (the lower) of their lines, while another group of participants was instructed to select the line that produced an *ascending* (a descending) sequence with the line selected by the co-actors when the co-actor selected the *lower* (the higher) of their lines. As in Experiment 1, in all four groups, selecting the correct line could require participants to adopt the same (congruent) or the opposite (incongruent) movement goal as the co-actor (i.e., selecting the left or the right of their lines). Thus Experiment 2 had the same 2 (Task Goal: Individual vs. Joint) × 2 (Action Goal Congruency: Congruent vs. Incongruent) × 2 (Movement Goal Congruency: Congruent vs. Incongruent) mixed factorial design as Experiment 1, with Task Goal and Action Goal Congruency manipulated between and Movement Goal Congruency manipulated within subjects.

#### 3.1.5. Data analysis

The data analysis procedure was identical to Experiment 1. 1.2% of all trials in the test phase were removed due to response omissions.

### 3.2. Results

**Figure 4:**
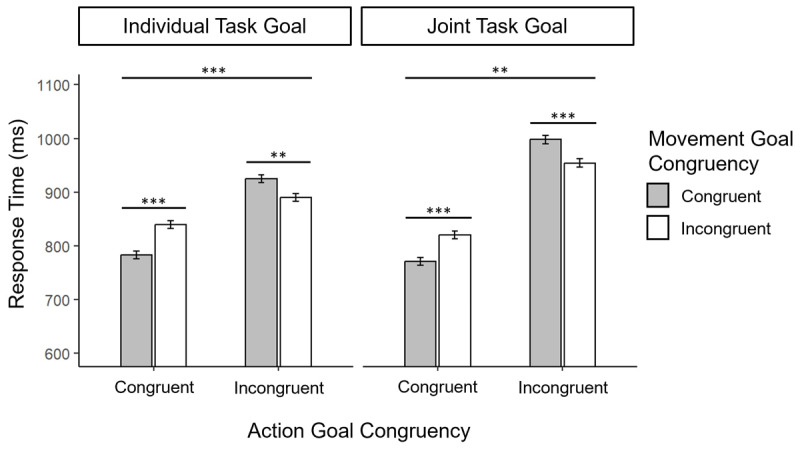
Response times in Experiment 2 as a function of Task Goal, Individual Goal Congruency and Response Congruency. Error bars represent standard errors of the mean (SEM) corrected for within subjects designs (Morey, 2008). Asterisks denote significant simple-effects contrasts between factor levels, conditional on the levels of the other factors. ***p < .001, **p < .01.

The results of the RT analysis of Experiment 2 is depicted in Figure 4.

Analysis of participants’ RTs revealed a significant main effect of Action Goal Congruency, *F*(1, 184) = 33.7, *p* < .001, η_p_^2^ = .16, BF_Inclusion_ = 207687, with faster RTs in the congruent action goal (mean = 803 ms, 95% CI [771 ms, 836 ms]) compared to the incongruent action goal conditions (mean = 942 ms, 95% CI [907 ms, 976 ms]). Yet, as in Experiment 1 the interaction between Action Goal Congruency and Task Goal was still off statistical significance, *F*(1, 184) = 3.11, *p* = .08, η_p_^2^ = .02, BF_Inclusion_ = 0.81, indicating no conclusive evidence for a moderating effect of Task Goal on the Action Goal Congruency effect. If anything, the RT difference between the action goal congruent and action goal incongruent conditions was descriptively larger in the Joint Task Goal condition (absolute mean difference = 181 ms, 95% CI [86.4 ms, 259 ms]) compared to the Individual Task Goal condition (absolute mean difference = 96.4 ms, 95% CI [2.31 ms, 191 ms]). Yet, similar to Experiment 1, results of Experiment 2 showed a significant interaction effect between Action Goal Congruency and Movement Goal Congruency, *F*(1, 184) = 76.9, *p* < .001, η_p_^2^ = .29, BF_Inclusion_ = 4.4 × 10^12^. Follow-up analysis of simple main effects showed that this interaction was due to a significant effect of Movement Goal Congruency in the Action Goal Congruent conditions, *F*(1, 96) = 51.4, *p_Bonf_* < .001, η_p_^2^ = .35, with faster RTs in trials with congruent movement goals (mean = 777 ms, 95% CI [766 ms, 787 ms]) compared to incongruent movement goals (mean = 830 ms, 95% CI [819 ms, 840 ms]) and a significant but *reversed* effect of Movement Goal Congruency in the Action Goal Incongruent conditions, *F*(1, 90) = 27.9, *p_Bonf_* < .001, η_p_^2^ = .24, with slower RTs in trials with congruent movement goals (mean = 961 ms, 95% CI [951 ms, 971 ms]) compared to incongruent movement goals (mean = 922 ms, 95% CI [912 ms, 932 ms]). The interaction between Action and Movement Goal Congruency was not further qualified by Task Goal (*F* < 1, BF_Inclusion_ = 0.278 for the three-way interaction term).

In line with the frequentist analysis, Bayesian model comparison revealed that the factor model including main effects of Action and Movement Goal Congruency as well as their interaction provided the best fit to the data (BF_M_ = 11.8). This model was favoured over the model that further included the interaction between Task Goal and Action Goal Congruency (BF_01_ = 1.91), indicating weak evidence against a moderating influence of Task Goal on the Action Goal Congruency effect. The best fitting model was also favoured over the model that included all further interactions up to the three-way interaction between all three design factors (BF_01_ = 25.8), indicating strong evidence against the hypothesis that the interaction between Action and Movement Goal Congruency was further moderated by participants Task Goal. A table with all Bayesian model comparisons is presented in Appendix B. Using different prior specifications for parameter values in each model produced qualitatively similar result patterns (result tables for Bayesian model comparisons with different prior options can be found in the supplement).

Results of the ER analysis were largely congruent with the RT analysis. Yet, besides a significant main effect of Action Goal Congruency, *F*(1, 184) = 8.51, *p* = .004, η_p_^2^ = .044, and a significant interaction between Action Goal Congruency and Movement Goal Congruency, *F*(1, 184) = 16.6, *p* < .001, η_p_^2^ = .083, we also found a significant main effect of Task Goal, *F*(1, 184) = 4.56, *p* = .034, η_p_^2^ = .024, which was driven by higher error rates in the joint (mean = 4.7%, 95% CI [3.9%, 5.5%]) compared to the individual task goal condition (mean = 3.4%, 95% CI [2.6%, 4.3%]). The detailed results of the ER analysis are presented in Appendix B.

### 3.3. Comparison between experiments

To evaluate whether the novel stimuli set used in Experiment 2 had the intended effect to enhance overall task performance compared to Experiment 1, we compared participants’ average RTs and ERs between Experiment 1 and Experiment 2 by means of independent samples t-tests. RTs were significantly faster in Experiment 2 (mean = 870 ms, 95% CI [852 ms, 889 ms]) compared to Experiment 1 (mean = 1081 ms, 95% CI [1058 ms, 1104 ms]), *t*(387) = 10.1, *p* < .001, d = 1.04. Comparing ERs between Experiment 1 (mean = 3.3%, 95% CI [2.8%, 3.8%]) and Experiment 2 (mean = 4.1%, 95% CI [3.5%, 4.6%]) showed no significant difference, *t*(387) = 1.93, *p* = .054, d = 0.20.

### 3.4. Discussion

The results of Experiment 2 replicated the findings of Experiment 1 in a modified version of our task that involved perceptual rather than conceptual discrimination of action targets. We again found evidence for an imitative congruency effect at the level of action goals, which showed to modulate imitative congruency effects at the level of co-actors’ lower-level movement goals. Yet, although modifying the task compared to Experiment 1 enhanced overall task performance in Experiment 2, we still found no evidence for a modulatory effect of Joint vs. Individual Task Goal instructions on imitative congruency effects between co-actors’ individual action and/or movement goals. Interestingly though, participants performing the task with the instruction to complement the card selection of their partner (Joint Task Goal condition) conducted more response errors than participants performing the task with the instruction to match/mismatch the individual action goal of their co-actor (Individual Task Goal condition). This finding seems to indicate that implementing the task instructions for selecting a response in the joint task goal condition appeared to be more difficult than selecting a response based on the instructions of the individual task goal condition.

## 4. General Discussion

In two experiments, we investigated whether and how higher-level goals to which own and others’ actions are individually or jointly directed modulate effects of imitative congruency between observed and self-executed contributions to a social interaction. More specifically, we asked 1) whether imitative congruency between co-actors’ individual action goals modulate imitative congruency effects between co-actors’ lower-level movement goals and 2) if acting towards joint as opposed to individual task goals ads to the control of imitative response tendencies in social interactions.

Results of both experiments showed evidence for an imitative congruency effect at the level of co-actors’ action goals which was found to modulate the impact of imitative congruency between co-actors’ lower-level movement goals. Yet, our findings provided no evidence that instructing participants to act towards joint rather than individual task goals modulated imitative congruency effects any further. In the following, we will discuss the implications of these findings in more detail.

### The influence of individual action goals on imitation

Regarding our first question, our findings provide additional evidence that imitative congruency effects between own and others’ lower-level movement goals become modulated by congruency relations between own and others’ higher-level action goals. Thus, our findings replicate earlier evidence for an interaction between higher-level action and lower-level movement goals in shaping imitative congruency effects in social interactions (Ondobaka et al., 2012, 2013). As such, our findings contrast with recent findings by Cole et al. (2018) and Janczyk et al. (2016), who found no indications for a modulation of movement goal congruency effects by co-actors’ higher-level action goals. However, in their task setups effects of movement goal congruency can be explained by attentional mechanisms (social inhibition of return, see Cole et al., 2019), which was ruled out in the current study by presenting co-actors’ action targets side-by-side rather than opposing each other. This design choice ensured that effects of movement goal congruency in the current study can be attributed to automatic imitation of observed movement goals rather than to low-level attentional mechanisms. The contrasting findings between the current study and those of Cole et al. (2018) and Janczyk et al. (2016), could thus be explained by differing mechanisms that produce movement goal congruency effects in the respective task setups. This suggests the conclusion that automatic imitation of observed movement goals but not lower-level attentional mechanisms (i.e., social inhibition of return) appear to be susceptible to modulations of co-actors’ higher-level action goals (but see, Schmitz et al., 2023).

Concerning mechanistic theories of imitation, our findings question the automaticity of people’s tendency to imitate low-level movement features of observed actions (e.g., Heyes, 2011; Rizzolatti et al., 2001). More specifically, our findings speak against the notion that imitation effects rely on direct matching between perceptual movement features of observed and self-executed actions, but suggest that low-level perception-action links can become flexibly adapted to current task demands (cf., van Schie et al., 2008). Our findings thus corroborate the hypothesis of an hierarchical control mechanism for imitative response tendencies in which higher-level representations of own and others’ actions in terms of their individual action goals determine the influence of lower-level representations of own and others’ actions in terms of their movement goals (Ondobaka & Bekkering, 2012, 2013). As such, our findings support goal-directed theories of imitation (Csibra, 2007; Wohlschläger et al., 2003) by showing that imitation effects are mainly determined by conceptual interpretations of own and others’ actions in terms of their higher-level action goals rather than by lower-level perceptual features of the movements people choose to reach them.

An important characteristic of our task setup that could have promoted the dominating role of individual action goals on imitation effects in the current study is that we restricted action observation to spatial features of a virtual co-actor’s action end state (signalled by the relative spatial location of their action target) instead of showing another person’s bodily movements towards one of the targets as well. Movement goal congruency was thus defined in terms of spatial compatibility between co-actors’ action targets and not by congruency between topographical features of their bodily effectors or movement trajectories. This aspect of our study suggests that the modulating effect of higher-level action goals on lower-level movement goal imitation found in the present study does not seem to be mediated by domain-specific mechanisms dedicated to the processing of others’ bodily movements, but might be rather attributed to domain-general mechanisms that guide action planning and control processes across social and non-social task settings.

For example, our findings could be an instance of reversed spatial stimulus-response compatibility effects that have been shown to arise when participants receive incongruent mapping instructions on a task relevant stimulus dimension (e.g., respond to red stimuli pressing a green key and vice versa) (Hedge & Marsh, 1975; Li et al., 2015; Proctor & F. Pick, 2003; Wühr & Biebl, 2009). This effect is commonly explained by logical recoding of the primary task rule (i.e., identity vs. reversal) being applied to task irrelevant stimulus dimensions as well. In the context of the present study, instructions to match/mismatch the action goal of a co-actor may have been recoded as instructions to “do the same/opposite”, which could have been inadvertently applied to task-irrelevant features of the co-actor’s action — here, the relative spatial position of their selected target, i.e., movement goals in our terminology — as well, resulting in the observed cross-over interaction pattern.

Furthermore, our findings could also be explained by short-term binding and retrieval processes (c.f., Frings et al., 2020 for an overview). It has been shown that partial repetitions of target and response features across consecutive trials (e.g., the target repeats but requires a different response than before) impair performance compared to complete repetitions or complete alternations (Frings et al., 2007; Hommel, 1998; Rothermund et al., 2005). Interestingly, these effects can also be observed when the previous trial is performed by another agent (Giesen et al., 2014, 2017; Giesen & Frings, 2021). Observing the target selection of the virtual partner in the present task setup may thus bind features of the target (high/low) with features of the observed response (left/right). This would interfere with participants subsequent target selection when only one but not both features alternate or repeat, which could explain the cross-over interaction pattern between action and movement goal congruency found in the present study.

Against this background, a relevant question for future research would be to which extend domain-general (e.g., logical recoding or feature binding) and domain-specific processes (e.g., motor simulation of observed movements) contribute to the control of imitative behaviour and how these processes interact with each other (see Michael & D’Ausilio, 2015; Ramsey & Ward, 2020; Ward & Ramsey, 2024 for initial proposals).

### The influence of joint task goals on imitation

Turning to our second question, our study revealed no evidence that instructing participants to work towards joint rather than individual task goals further modulated imitative congruency effects emerging between co-actors’ individual action and/or movement goals. In particular, instructing participants to produce joint action outcomes by complementing their co-actor target selection was not found to alter an evident tendency of participants to imitate the individual action goal of their co-actor, nor the modulating influence of action goal congruency on imitative congruency effects between co-actors’ movement goals. This finding is noteworthy, as performing the task in the joint task goal condition as instructed did not require participants explicitly to adopt the same or opposite individual action goal as the co-actor (i.e., selecting the higher or the lower of their targets) but to complement their co-actors’ target selection in the service of a joint goal instead (i.e., selecting the target that produces and ascending or descending sequence with the target elected by the co-actor). The finding of similar imitation effects between the individual and joint task goal condition thus seem to contrast with previous research reporting evidence for a modulating effect of joint task goals on imitative congruency effects between co-actors’ individual action contributions (Clarke et al., 2019; Rocca et al., 2023; Sacheli et al., 2018). This raises the question, why imitative congruency effects between co-actors’ individual action and movement goals remained present in the current study even when participants were instructed to pursue a joint task goal together with their co-actor.

To explain modulations of imitative congruency effects by joint task goals, theories of joint action have suggested that representations of joint task goals lead co-actors to integrate separate representations of own and others’ individual action contributions into *hierarchical joint action plans* that also encode complementary relations between co-actors’ individual action contributions (Candidi et al., 2015; Marschner et al., 2024; Pesquita et al., 2018; Sacheli et al., 2018; Sebanz & Knoblich, 2021; Sinigaglia & Butterfill, 2022; Zapparoli et al., 2022). Our findings may thus indicate that participants in the joint task goal condition did not represent their own and their co-actors’ actions as complementary contributions to a joint goal they were pursuing together but rather as separate action contributions guided by individual action goals of selecting the higher or the lower of their targets instead. Although staying tentatively, we reason that this could be explained by specific task characteristics of the current study, imbalanced task demands between the individual and joint task goal condition or by a more general bias to encode own and others’ contributions to a joint action in terms of individual rather than joint goals.

Regarding task characteristics, one limitation of the current study is that our task involved computer-based interaction with a virtual co-actor in an online test setting. Yet, it is possible that adoption of joint task goals and integration of own and others’ actions into hierarchical joint action plans guided by joint rather than individual goal representations requires real-life embodied interaction with human co-actors (cf., Ciardo et al., 2022; Sahaï et al., 2019 for empirical findings; but see Strasser, 2015, 2018 for discussion). Thus, although simulating an interactive task with a virtual, disembodied co-actor proved to produce significant imitation effects in the current study, it could have been insufficient to trigger further integration of own and others’ actions into hierarchical joint action plans.

Moreover, in the current task setup, participants were always required to adapt their actions to those of their co-actor but never vice versa. Arguably, this introduced a leader-follower hierarchy to the task that prevented a need for mutual adaptation and common coordination requirements between both co-actors. Yet, mutual adaptation, common coordination requirements and egalitarian role distributions have been identified as critical task characteristics that lead co-actors to develop a sense of acting together as a group (Bolt et al., 2016; Le Bars et al., 2020, 2022; Pacherie, 2014) or to feel truly engaged in a meaningful social interaction (Di Paolo & De Jaegher, 2012). It is thus possible that unidirectional coordination demands on site of the participants made them reluctant to adopt the instructed joint task goal of producing joint outcomes together with their co-actor and made them more likely to encode their own action contributions in terms of the individual task goal of matching or mismatching the card selection of their co-actor instead.

Finally, another explanation for the presence of imitative congruency effects between co-actors individual action and movement goals under both task goal instructions could be that implementation of the joint task goal instructions confronted participants with unnecessary high task demands that could be circumvented by adopting an individual task goal instead (i.e., matching or mismatching the action goal of the co-actor by selecting one’s higher or one’s lower target). As mentioned earlier, this interpretation would be supported by action identification theory (Vallacher & Wegner, 1985, 2012), proposing that people tend to conceptualize their actions in a given task context in a way that ensures most efficient task performance. Thus, participants in the joint task goal condition may have found it more easy to perform the task in an efficient way by discarding the joint task goal instruction of building ascending and descending target sequences and by adopting an individual task goal of matching or mismatching the target selection of their co-actors instead. This interpretation would also be in line with the finding of higher error rates in the joint compared to the individual task goal condition in Experiment 2 which may reflect difficulties with initial or partial attempts to implement the instructions of the joint task goal conditions.^5^

This interpretation would suggest that representing own and others’ contributions to a shared task as integrated complementary contributions towards a joint goal is not necessarily a default strategy for joint action planning but may be rather dependent on its utility for guiding efficient action performance in a given task context. Indeed, it has been proposed that joint action planning *minimally* requires co-actors to represent their joint goal as well as their own task contribution needed to achieve it while only being aware that the joint goal cannot be achieved by acting alone (Vesper et al., 2010). Such minimal joint action representations would thus not require that co-actors integrate representations of their own and their partners’ individual action contributions into more elaborated hierarchical joint action plans that encode complementary relations between co-actors’ individual task contributions. Yet, they might be often sufficient to guide efficient action performance in a given task. One possibility suggested by our findings could thus be that co-actors default to represent interactive tasks by means of action plans that remain limited on separate representations of their own and their partners’ individual action contributions (i.e., individual action or movement goals), while additional efforts to further represent complementary relations between individual action contributions of self and others into hierarchical joint action plans may become only engaged when beneficial for current task performance. A promising direction for future research on joint action planning could thus be to identify conditions and enabling factors that provoke co-actors to switch from action plans centred at individual action and movement goals to more elaborated hierarchical representations of joint action that also encode complementary relations between co-actors’ individual action and/or movement goals as well.

## 5. Conclusion

In summary, the present study provides complementing evidence that tendencies to imitate low-level movement features of observed actions are modulated by conceptual interpretations of own and others’ actions in terms of their higher-level individual action goals. While this finding supports goal-directed theories of imitation, our study further suggests that the modulating influence of higher-level individual action goals on imitative action tendencies may arise from domain-general action planning and control mechanisms that operate across social and non-social tasks settings. Furthermore, our study indicates that explicit instructions to pursue joint rather than individual task goals are limited in their ability to modulate imitative congruency effects between co-actors’ individual contributions to a joint task. This finding suggests that action planning in interactive task contexts may often remain organized around representations of co-actors’ individual-level action and/or movement goals, while relational integration of own and others’ actions into hierarchical joint action plans may only become engaged when afforded by current task demands.

## Data Accessibility Statement

All experimental materials, raw data and analysis scripts are available at https://osf.io/wc96q/?view_only=dd5091015d744e81a0295afdb0050d49.

## Additional File

The additional file for this article can be found as follows:

10.5334/joc.483.s1 Supplementary File. Tables S1–S4.

## Figures and Tables

**Figure A1:**
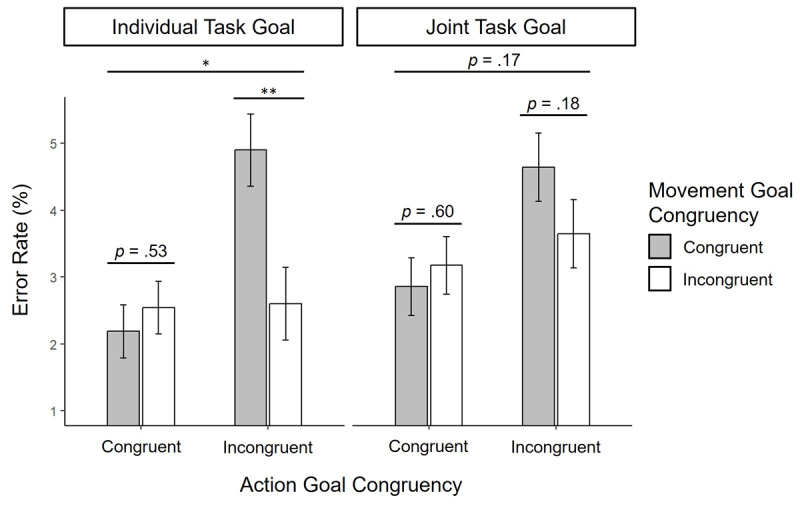
Mean error rates in Experiment 1 as a function of Task Goal, Action Goal Congruency and Movement Goal Congruency. Error bars represent standard errors of the mean (SEM) corrected for within subjects designs (Morey, 2008). Asterisks denote significant simple-effects contrasts between factor levels, conditional on the remaining factors; numeric labels indicate exact p-values for non-significant contrasts. **p < .01, *p < .05.

**Figure B1:**
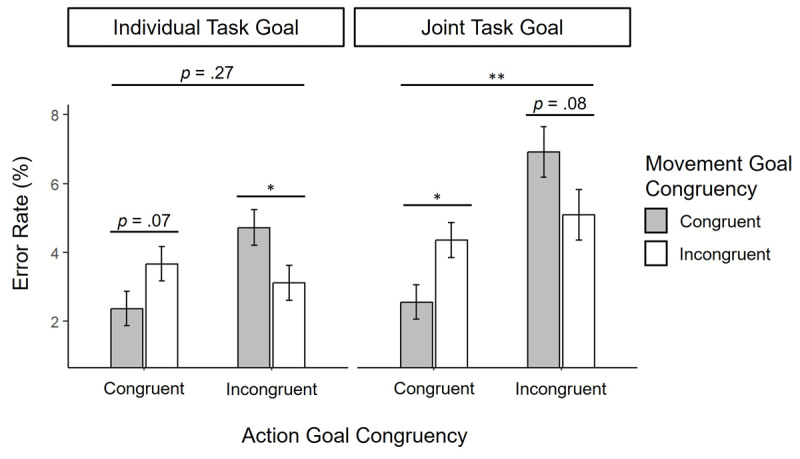
Error rates in Experiment 2 as a function of Task Goal, Action Goal Congruency and Movement Goal Congruency. Error bars represent standard errors of the mean (SEM) corrected for within subjects designs (Morey, 2008). Asterisks denote significant simple-effects contrasts between factor levels, conditional on the remaining factors; numeric labels indicate exact p-values for non-significant contrasts. **p < .01, *p < .05.

## Footnotes

Note that the two cards of the participant always showed the next higher and the next lower card compared to the card selected by the co-actor. This ensured that selecting one of the two cards completes an ascending card sequence while selecting the other card completes a descending number sequence.

Note that, as such, participants could have principally performed the individual task goal condition by following the instructions of the joint task goal condition and vice versa. As this design feature makes carry over effects between different blocks manipulating Task Goal within subjects highly likely, Task Goal was manipulated as a between-subjects factor.

In both experiments reported, participants with inadequate training performance had to quit the experiment and were compensated with 1 GBP for their time spent on the study. The final sample size only included participants who completed the whole experiment.

Confidence intervals for within-subjects contrasts are adjusted for within-subjects designs (Morey, 2008).

As suggested by an anonymous reviewer, an alternative explanation of this finding could be that the joint task goal condition could have led to a parallel activation of joint and individual goal representations (Sartori et al., 2015), increasing cognitive demands in the joint compared to the individual task goal condition. Note that this interpretation would also be in line with our suggestion that participants may have coped with increased cognitive demands in the joint task goal condition by adopting an individual task goal instead.

## Appendix A – Additional results for Experiment 1

**Table A1:** Bayesian Model Comparison Results for Response Time Analysis in Experiment 1.

MODELS	P(M)	P(M|DATA)	BF_M_	BF_01_	ERROR %

Movement Goal Congruency + Action Goal Congruency + Movement Goal Congruency × Action Goal Congruency	0.053	0.338	9.201	1.000	

Action Goal Congruency	0.053	0.193	4.293	1.757	3.029

Movement Goal Congruency + Action Goal Congruency + Task Goal + Movement Goal Congruency × Action Goal Congruency	0.053	0.169	3.658	2.003	8.692

Movement Goal Congruency + Action Goal Congruency + Task Goal + Movement Goal Congruency × Action Goal Congruency + Action Goal Congruency × Task Goal	0.053	0.086	1.683	3.956	10.217

Action Goal Congruency + Task Goal	0.053	0.084	1.656	4.016	6.297

Action Goal Congruency + Task Goal + Action Goal Congruency × Task Goal	0.053	0.048	0.905	7.064	6.754

Movement Goal Congruency + Action Goal Congruency	0.053	0.026	0.481	12.991	17.356

Movement Goal Congruency + Action Goal Congruency + Task Goal + Movement Goal Congruency × Action Goal Congruency + Movement Goal Congruency × Task Goal	0.053	0.024	0.439	14.220	11.878

Movement Goal Congruency + Action Goal Congruency + Task Goal + Movement Goal Congruency × Action Goal Congruency + Movement Goal Congruency × Task Goal + Action Goal Congruency × Task Goal	0.053	0.012	0.210	29.304	10.631

Movement Goal Congruency + Action Goal Congruency + Task Goal	0.053	0.009	0.170	36.113	7.868

*Note*. Only the 10 best out of all 19 models are shown. Results were obtained using a default prior specification for model parameters with *r* scale for fixed effects = .5. Model comparison results using two different prior settings can be found in the supplement.

**Table A2:** ANOVA Results for Error Rate Analysis in Experiment 1.

EFFECT	*DF*	*F*	*P*	η_P_^2^

Task Goal	1,188	1.105	.295	.006

Action Goal Congruency	1,188	6.349	.013	.033

Movement Goal Congruency	1,188	3.821	.052	.02

Task Goal × Action Goal Congruency	1,188	0.065	.799	3.4 × 10^–4^

Task Goal × Movement Goal Congruency	1,188	0.898	.345	.005

Action Goal Congruency × Movement Goal Congruency	1,188	8.760	.003	.045

Task Goal × Action Goal Congruency × Movement Goal Congruency	1,188	1.002	.318	.005

*Note*. Based on Type III Sum of Squares.

## Appendix B – Additional Results for Experiment 2

**Table B1:** Bayesian Model Comparison Results for Response Time Analysis in Experiment 2.

MODELS	P(M)	P(M|DATA)	BF_M_	BF_01_	ERROR %

Movement Goal Congruency + Action Goal Congruency + Movement Goal Congruency × Action Goal Congruency	0.053	0.397	11.841	1.000	

Movement Goal Congruency + Action Goal Congruency + Task Goal + Movement Goal Congruency × Action Goal Congruency	0.053	0.272	6.733	1.458	8.974

Movement Goal Congruency + Action Goal Congruency + Task Goal + Movement Goal Congruency × Action Goal Congruency + Action Goal Congruency × Task Goal	0.053	0.207	4.710	1.913	6.339

Movement Goal Congruency + Action Goal Congruency + Task Goal + Movement Goal Congruency × Action Goal Congruency + Movement Goal Congruency × Task Goal + Action Goal Congruency × Task Goal	0.053	0.055	1.053	7.181	11.928

Movement Goal Congruency + Action Goal Congruency + Task Goal + Movement Goal Congruency × Action Goal Congruency + Movement Goal Congruency × Task Goal	0.053	0.052	1.006	7.495	10.774

Movement Goal Congruency + Action Goal Congruency + Task Goal + Movement Goal Congruency × Action Goal Congruency + Movement Goal Congruency × Task Goal + Action Goal Congruency × Task Goal + Movement Goal Congruency × Action Goal Congruency × Task Goal	0.053	0.015	0.281	25.787	33.670

Movement Goal Congruency	0.053	3.5 × 10^–13^	6.3 × 10^–12^	1.1 × 10^12^	4.041

Action Goal Congruency + Task Goal + Action Goal Congruency × Task Goal	0.053	2.4 × 10^–13^	4.0 × 10^–12^	1.6 × 10^12^	6.261

Action Goal Congruency + Task Goal	0.053	1.9 × 10^–13^	3.3 × 10^–12^	2.1 × 10^12^	6.165

Movement Goal Congruency + Action Goal Congruency	0.053	9.4 × 10^–14^	1.7 × 10^–12^	4.2 × 10^12^	4.011

*Note*. Only the 10 best out of all 19 models are shown. Results were obtained using a default prior specification for model parameters with *r* scale for fixed effects = .5. Model comparison results using two different prior settings can be found in the supplement.

**Table B2:** ANOVA Results for Error Rate Analysis in Experiment 2.

EFFECT	*DF*	*F*	*P*	η_P_^2^

Task Goal	1,184	4.562	.034	.024

Action Goal Congruency	1,184	8.505	.004	.044

Movement Goal Congruency	1,184	0.044	.834	2.4 × 10^–4^

Task Goal × Action Goal Congruency	1,184	1.939	.166	.010

Task Goal × Movement Goal Congruency	1,184	0.027	.869	1.4 × 10^–4^

Action Goal Congruency × Movement Goal Congruency	1,184	16.632	< .001	.083

Task Goal × Action Goal Congruency × Movement Goal Congruency	1,184	0.206	.651	.001

*Note*. Based on Type III Sum of Squares.
