# Supplementary material for: Do Individual and Joint Action Goals Modulate Imitative Response Tendencies?
Source: J Cogn. 2026 Jan 9;9(1):10. doi: 10.5334/joc.483 (PMC12785670; doi:10.5334/joc.483)
Supplement: Supplementary File. — Tables S1–S4. [file joc-9-1-483-s1.pdf]

Table S1

Bayesian Model Comparison Results for Response Time Analysis in Experiment 1; r scale for fixed effects = .2

Models	P(M)	P(M|data)	BF_M	BF_{01}	error %
Movement Goal Congruency + Action Goal Congruency + Movement Goal Congruency × Action Goal Congruency	0.053	0.274	6.789	1.000	
Movement Goal Congruency + Action Goal Congruency + Task Goal + Movement Goal Congruency × Action Goal Congruency	0.053	0.221	5.116	1.238	11.43
Movement Goal Congruency + Action Goal Congruency + Task Goal + Movement Goal Congruency × Action Goal Congruency + Action Goal Congruency × Task Goal	0.053	0.176	3.853	1.553	12.79
Action Goal Congruency	0.053	0.074	1.433	3.713	15.63
Movement Goal Congruency + Action Goal Congruency + Task Goal + Movement Goal Congruency × Action Goal Congruency + Movement Goal Congruency × Task Goal	0.053	0.066	1.274	4.144	14.31
Movement Goal Congruency + Action Goal Congruency + Task Goal + Movement Goal Congruency × Action Goal Congruency + Movement Goal Congruency × Task Goal + Action Goal Congruency × Task Goal	0.053	0.045	0.846	6.104	17.13
Action Goal Congruency + Task Goal	0.053	0.041	0.769	6.686	10.00
Movement Goal Congruency + Action Goal Congruency + Task Goal + Movement Goal Congruency × Action Goal Congruency + Movement Goal Congruency × Task Goal + Action Goal Congruency × Task Goal + Movement Goal Congruency × Action Goal Congruency × Task Goal	0.053	0.030	0.550	9.235	16.21
Action Goal Congruency + Task Goal + Action Goal Congruency × Task Goal	0.053	0.029	0.536	9.465	10.79
Movement Goal Congruency + Action Goal Congruency	0.053	0.016	0.285	17.577	4.959

Note. Only the 10 best out of all 19 models are shown. Results were obtained using a default prior specification for model parameters with *r* scale for fixed effects = .2.

Table S2

Bayesian Model Comparison Results for Response Time Analysis in Experiment 1; r scale for fixed effects = .8

Models	P(M)	P(M|data)	BF_M	BF_{01}	error %
Action Goal Congruency	0.053	0.352	9.795	1.000	
Movement Goal Congruency + Action Goal Congruency + Movement Goal Congruency × Action Goal Congruency	0.053	0.311	8.131	1.133	3.211
Action Goal Congruency + Task Goal	0.053	0.106	2.141	3.315	5.798
Movement Goal Congruency + Action Goal Congruency + Task Goal + Movement Goal Congruency × Action Goal Congruency	0.053	0.101	2.014	3.501	8.011
Action Goal Congruency + Task Goal + Action Goal Congruency × Task Goal	0.053	0.040	0.753	8.773	6.550
Movement Goal Congruency + Action Goal Congruency + Task Goal + Movement Goal Congruency × Action Goal Congruency + Action Goal Congruency × Task Goal	0.053	0.038	0.714	9.238	8.279
Movement Goal Congruency + Action Goal Congruency	0.053	0.024	0.444	14.637	2.026
Movement Goal Congruency + Action Goal Congruency + Task Goal + Movement Goal Congruency × Action Goal Congruency + Movement Goal Congruency × Task Goal	0.053	0.010	0.182	35.269	9.485
Movement Goal Congruency + Action Goal Congruency + Task Goal	0.053	0.009	0.158	40.557	6.875
Movement Goal Congruency + Action Goal Congruency + Task Goal + Movement Goal Congruency × Action Goal Congruency + Movement Goal Congruency × Task Goal + Action Goal Congruency × Task Goal	0.053	0.004	0.067	94.887	10.998

Note. Only the 10 best out of all 19 models are shown. Results were obtained using a default prior specification for model parameters with *r* scale for fixed effects = .8.

Table S3

Bayesian Model Comparison Results for Response Time Analysis in Experiment 2; r scale for fixed effects = .2

Models	P(M)	P(M|data)	BF_M	BF_{01}	error %
Movement Goal Congruency + Action Goal Congruency + Movement Goal Congruency × Action Goal Congruency	0.053	0.522	19.621	1.000	
Movement Goal Congruency + Action Goal Congruency + Task Goal + Movement Goal Congruency × Action Goal Congruency	0.053	0.212	4.840	2.461	8.237
Movement Goal Congruency + Action Goal Congruency + Task Goal + Movement Goal Congruency × Action Goal Congruency + Movement Goal Congruency × Task Goal	0.053	0.177	3.860	2.953	23.27
Movement Goal Congruency + Action Goal Congruency + Task Goal + Movement Goal Congruency × Action Goal Congruency + Action Goal Congruency × Task Goal	0.053	0.057	1.093	9.114	9.504
Movement Goal Congruency + Action Goal Congruency + Task Goal + Movement Goal Congruency × Action Goal Congruency + Movement Goal Congruency × Task Goal + Action Goal Congruency × Task Goal	0.053	0.023	0.415	23.156	8.606
Movement Goal Congruency + Action Goal Congruency + Task Goal + Movement Goal Congruency × Action Goal Congruency + Movement Goal Congruency × Task Goal + Action Goal Congruency × Task Goal + Movement Goal Congruency × Action Goal Congruency × Task Goal	0.053	0.010	0.185	51.166	9.315
Action Goal Congruency	0.053	4.3×10^{-13}	7.8×10^{-12}	1.2×10^{12}	4.434
Movement Goal Congruency + Action Goal Congruency	0.053	2.4×10^{-13}	4.4×10^{-12}	2.2×10^{12}	10.67
Action Goal Congruency + Task Goal	0.053	2.2×10^{-13}	4.0×10^{-12}	2.4×10^{12}	23.72
Movement Goal Congruency + Action Goal Congruency + Task Goal	0.053	1.8×10^{-13}	3.2×12^{-12}	2.9×10^{12}	15.88

Note. Only the 10 best out of all 19 models are shown. Results were obtained using a default prior specification for model parameters with *r* scale for fixed effects = .2.

Table S4

Bayesian Model Comparison Results for Response Time Analysis in Experiment 2; r scale for fixed effects = .8

Models	P(M)	P(M|data)	BF_M	BF_{01}	error %
Movement Goal Congruency + Action Goal Congruency + Movement Goal Congruency × Action Goal Congruency	0.053	0.518	19.340	1.000	
Movement Goal Congruency + Action Goal Congruency + Task Goal + Movement Goal Congruency × Action Goal Congruency	0.053	0.228	5.328	2.268	24.03
Movement Goal Congruency + Action Goal Congruency + Task Goal + Movement Goal Congruency × Action Goal Congruency + Action Goal Congruency × Task Goal	0.053	0.196	4.375	2.649	23.96
Movement Goal Congruency + Action Goal Congruency + Task Goal + Movement Goal Congruency × Action Goal Congruency + Movement Goal Congruency × Task Goal	0.053	0.030	0.552	17.403	25.16
Movement Goal Congruency + Action Goal Congruency + Task Goal + Movement Goal Congruency × Action Goal Congruency + Movement Goal Congruency × Task Goal + Action Goal Congruency × Task Goal	0.053	0.025	0.459	20.847	23.54
Movement Goal Congruency + Action Goal Congruency + Task Goal + Movement Goal Congruency × Action Goal Congruency + Movement Goal Congruency × Task Goal + Action Goal Congruency × Task Goal + Movement Goal Congruency × Action Goal Congruency × Task Goal	0.053	0.004	0.063	147.570	23.66
Action Goal Congruency	0.053	$4.5×10^{-13}$	$8.0×10^{-12}$	$1.2×10^{12}$	22.97
Action Goal Congruency + Task Goal + Action Goal Congruency × Task Goal	0.053	$1.8×10^{-13}$	$3.3×10^{-12}$	$2.9×10^{12}$	22.98
Action Goal Congruency + Task Goal	0.053	$1.8×10^{-13}$	$3.2×10^{-12}$	$2.9×10^{12}$	22.91
Movement Goal Congruency + Action Goal Congruency + Task Goal	0.053	$7.6×10^{-14}$	$1.4×12^{-12}$	$6.8×10^{12}$	22.95

Note. Only the 10 best out of all 19 models are shown. Results were obtained using a default prior specification for model parameters with *r* scale for fixed effects = .8.
